# Supplementary material for: Social hierarchy influences monkeys’ risky decisions
Source: Commun Biol. 2026 Mar 11;9:578. doi: 10.1038/s42003-026-09817-2 (PMC13111692; doi:10.1038/s42003-026-09817-2)
Supplement: Supplementary file 1 — Supplementary Information [file 42003_2026_9817_MOESM1_ESM.pdf]

Supporting Information for

Social hierarchy influences monkeys' risky decisions

Naomi Chaix-Eichel[1,2,3†], Ayrton Guerillon[4,5†], Sacha Bourgeois-Gironde[6,7,8], Nicolas P. Rougier[1,2,3††], Thomas Boraud[1,9††], Sébastien Ballesta[4,5††*]
*Sébastien Ballesta
Email: ballesta@unistra.fr

This PDF file includes:

Supplementary Figures 1-10
Supplementary Tables 1-3

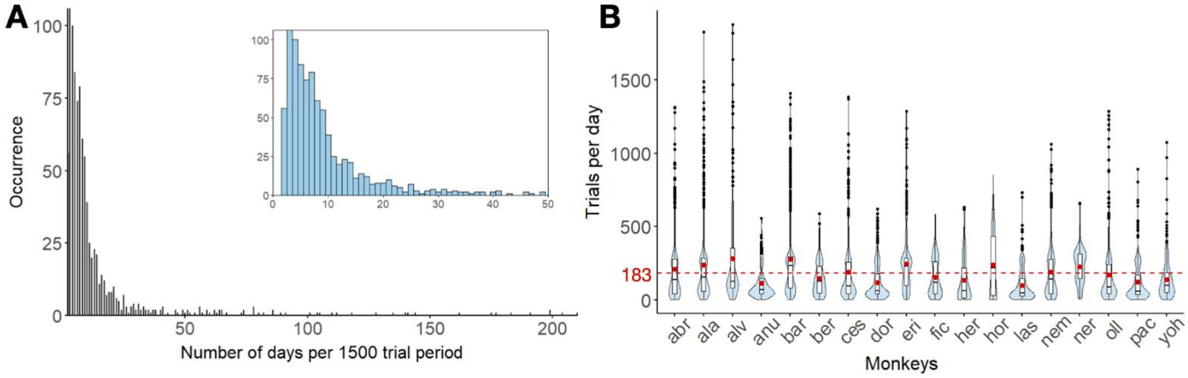

Supplementary Figure 1. (A) Distribution of the number of days within a 1500-trial period. For clarity, the x-axis is limited to 50 days in the blue panel, which encompasses approximately 95% of all periods included in the analyses. Periods shorter than 10 days account for 66.34% of all periods analyzed. **(B) Distribution of the number of trials per day.** Red points indicate the mean number of trials per day for each individual. Fewer than 50 trials per day represent 31.80% of the dataset, fewer than 100 trials account for 46.88%, fewer than 250 trials for 71.75%, fewer than 500 trials for 92.69%, and fewer than 1000 trials for 98.92% of the dataset.

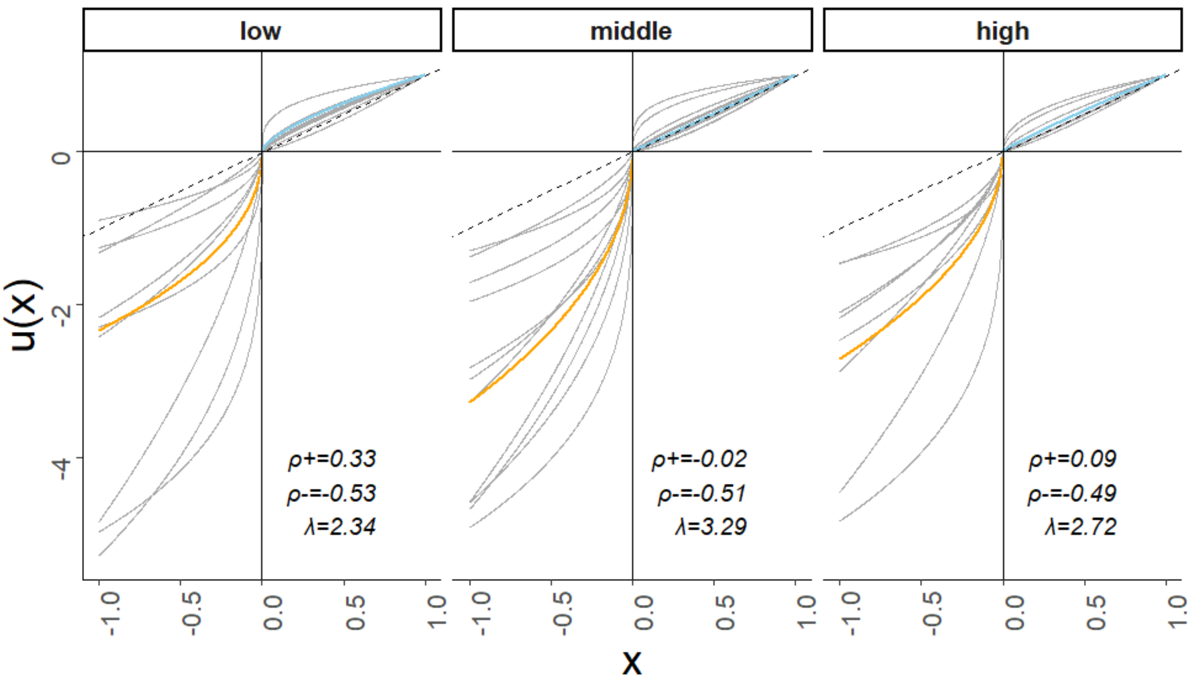

Supplementary Figure 2. Utility functions illustrating risk attitudes across domains for three Elo-score categories. Monkeys are grouped by Elo score: high (left), middle (center), and low (right). Elo ratings were categorized by dividing the overall range of the group's Elo scores into three equal tiers: low (584.12 to 835.81), middle (835.81 to 1087.50), and high (1087.50 to 1339.19). The blue curve represents the mean utility in the gain domain, while the orange curve represents the loss domain. Black curves depict individual utility functions. Overall, the utility function is concave in the gain domain, reflecting general risk aversion, and convex in the loss domain, indicating risk seeking. The steeper slope in the loss domain compared to the gain domain highlights an overall tendency toward loss aversion. Mean parameter values are indicated in the bottom-left corner.

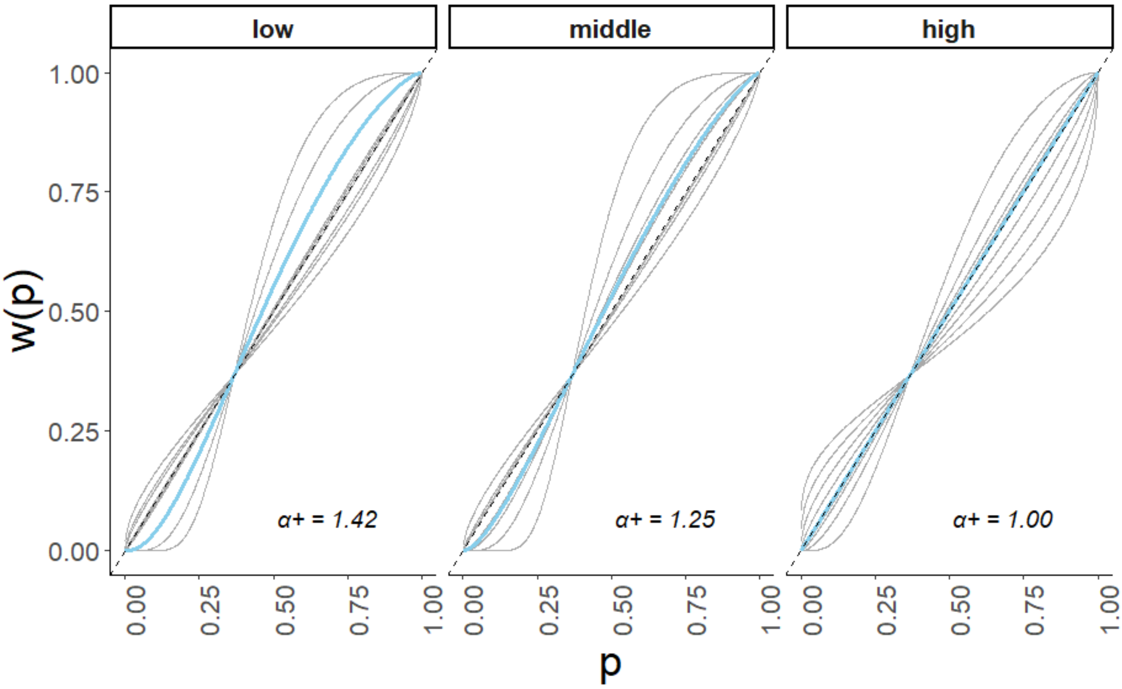

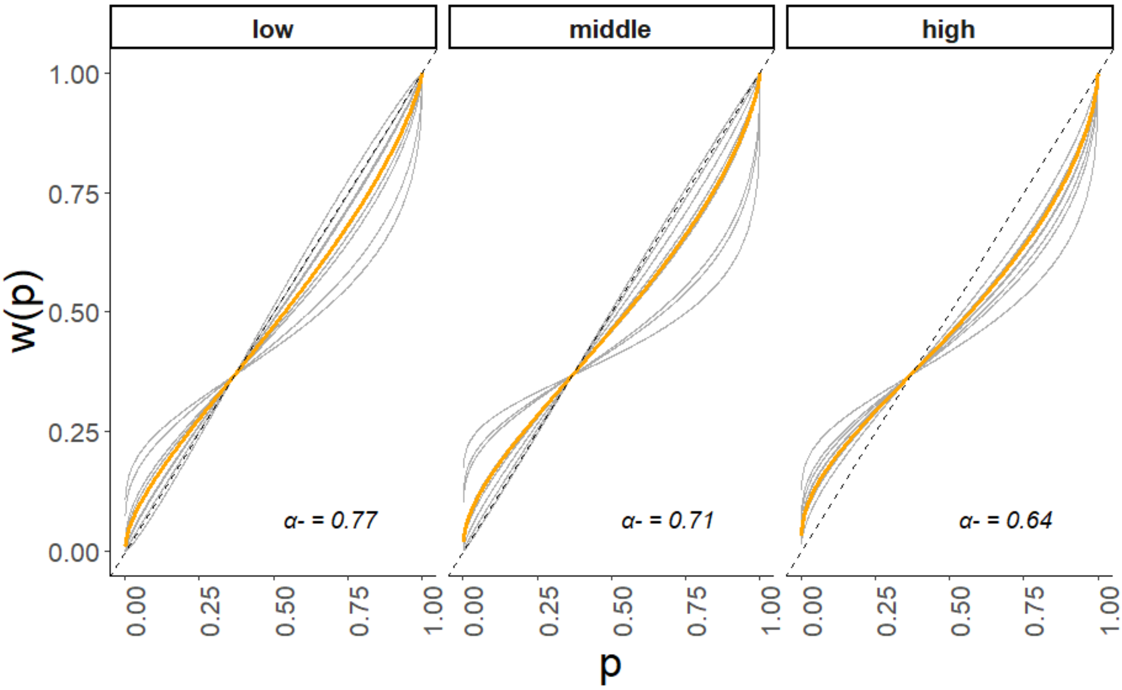

Supplementary Figure 3. Probability weighting function across domains for three Elo-score categories. Monkeys are grouped by Elo score: high (left), middle (center), and low (right). Elo ratings were categorized by dividing the overall range of the group's Elo scores into three equal tiers: low (584.12 to 835.81), middle (835.81 to 1087.5), and high (1087.5 to 1339.19). Blue and orange curves represent overall gain and loss probability distortion respectively while grey curves represent the mean probability distortion for each individual. Top: The blue curves represent probability weighting function for gain. The distortion parameter α_+ is superior to 1 indicating a global and overall, under estimation of low

probabilities and over estimation of high probabilities for gains. Bottom: The orange curves represent probability weighting function for gain. The distortion parameter α_- is inferior to 1 indicating a global and over estimation of low probabilities and under estimation of high probabilities for losses.

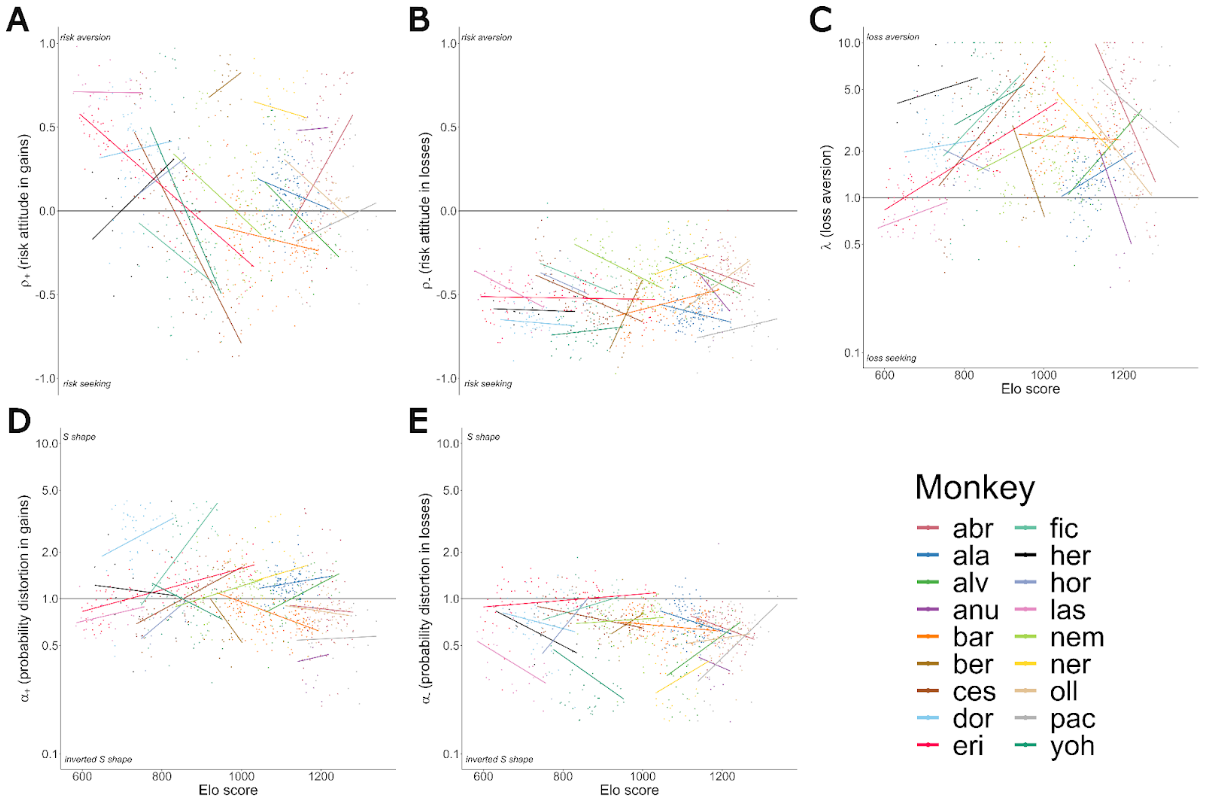

Supplementary Figure 4. Scatter plots showing the relationship between ρ, λ and α parameters and Elo-rating in gains and losses with linear regression for each individual. The quadratic effects of social hierarchy identified in the LMM cannot be observed at the individual level, as individuals would have to navigate from the bottom to the top of the group hierarchy (or vice versa) to be able to observe it, which did not happen in the group of monkeys we studied. However, the overall variations of the slopes of the individual linear relationships of PT parameters with Elo-rating seems to be consistent with the identified U-shaped effect in the LMM (Panels A, C and D only). A logarithmic transformation has been applied to the α and λ axes to normalize the representation of behavioral variations, as changes in values between 0.1-1 and 1-10 reflect opposite but equivalent intensities of behavior.

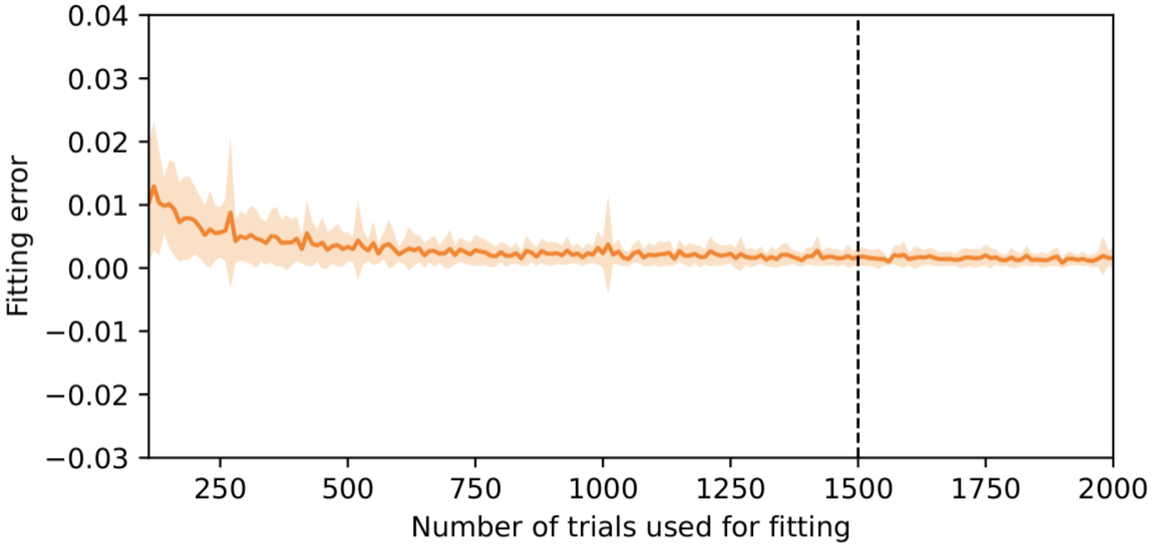

Supplementary Figure 5. Evolution of the fitting error score for PT parameters as the number of trials used to do the fit. After 1500 trials (any type of lotteries), the error does not decrease significantly. This measure has been averaged over 25 players (using random selection of value for the parameters).

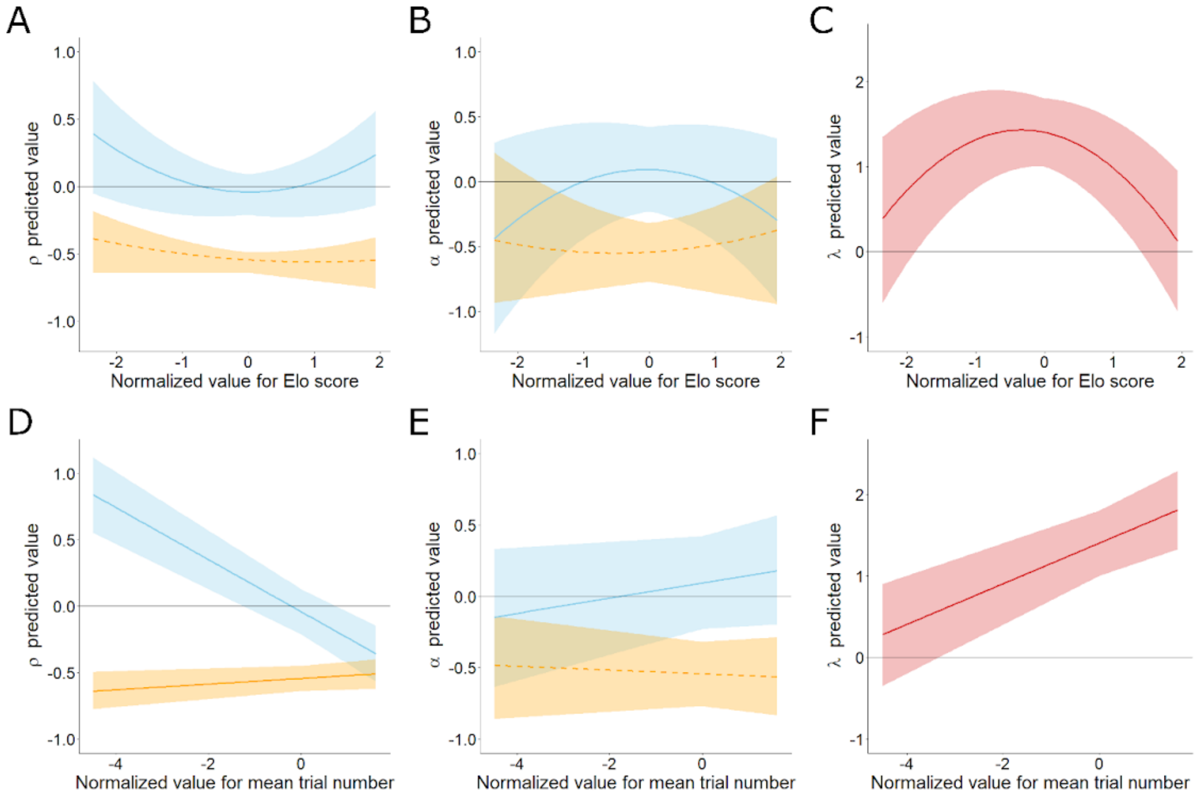

Supplementary Figure 6. LMM predictions for ρ and α as a function of Elo score and mean trial number. (A). Predicted values for ρ for gains (blue) and losses (orange) as a function of Elo score. **(B).** Predicted values for α for gains and losses as a function of Elo score. **(C).** Predicted values for λ as a function of Elo score. **(D).** Predicted values for ρ for gains and losses as a function of mean trial number, a proxy of subject experience in the task. **(E).** Predicted values for α for gains and losses as a function of mean trial number. **(F).** Predicted values for λ as a function of mean trial number. In all panels, predicted values parameters ρ and α are plotted separately for the gains condition (blue) and the losses condition (orange). The shaded areas around the regression lines represent the 95% confidence intervals, providing an estimate of the uncertainty around the predicted values. Estimates and CI are provided by LMM from table 1. Solid lines indicate statistically significant effects ($p < 0.01$), while dashed lines indicate non-significant effects ($p > 0.01$).

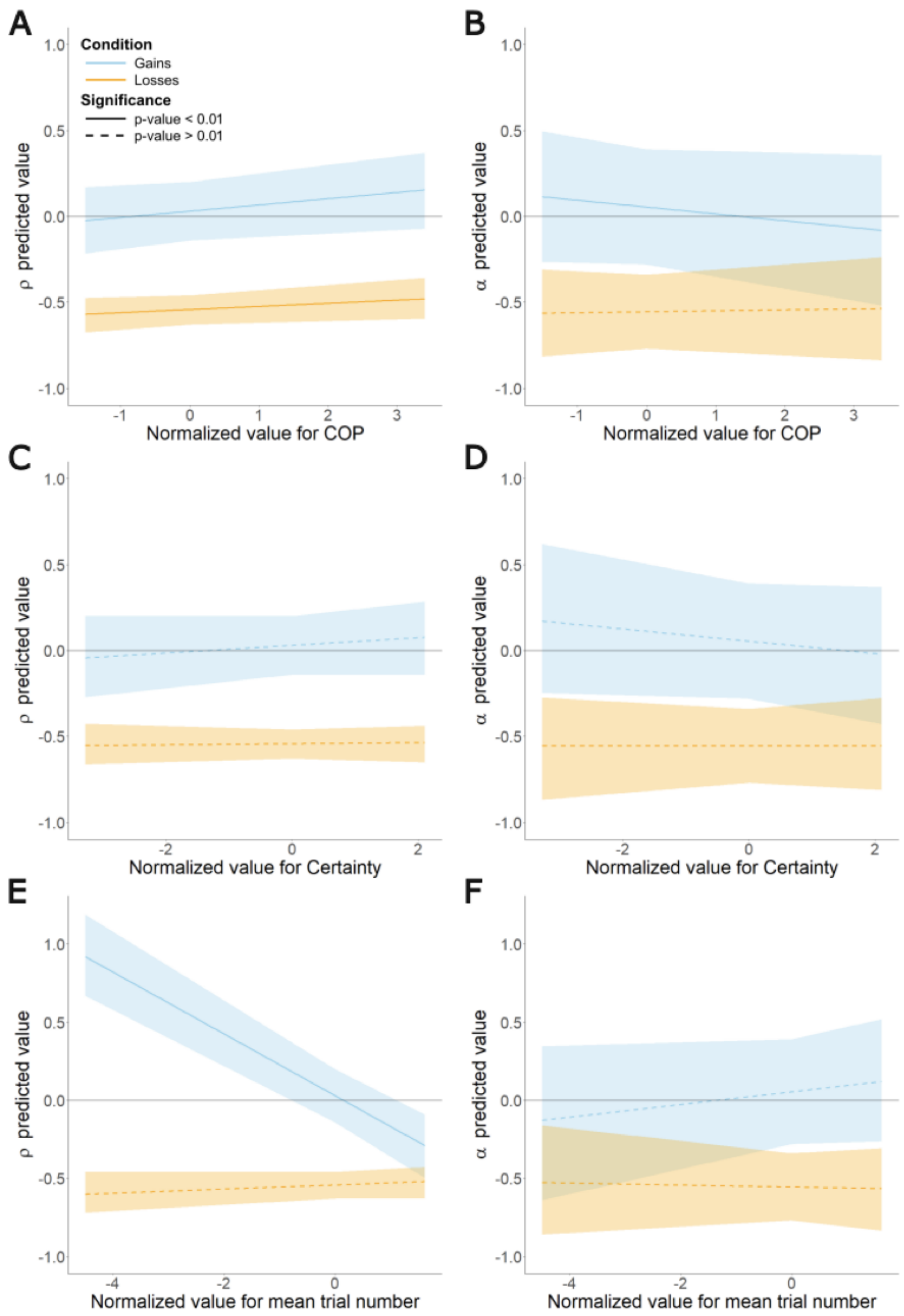

Supplementary Figure 7. Results of the linear mixed model predicting ρ and α as a function of COP, Certainty and mean trial number. (A, C E) show the predicted values for ρ, while (B, D, F) show the predicted values for α. The x-axis represents the normalized values of COP (A, B), Certainty (C, D) and mean trial number (E, F). The predicted values of ρ and α are plotted separately for the gains condition (blue) and the losses condition (orange). Solid lines indicate statistically significant effects ($p < 0.01$), while dashed lines indicate non-significant effects ($p > 0.01$). The shaded areas around the regression lines represent the 95%

confidence intervals, providing an estimate of the uncertainty around the predicted values. Estimates and CI are provided by LMM from table S1.

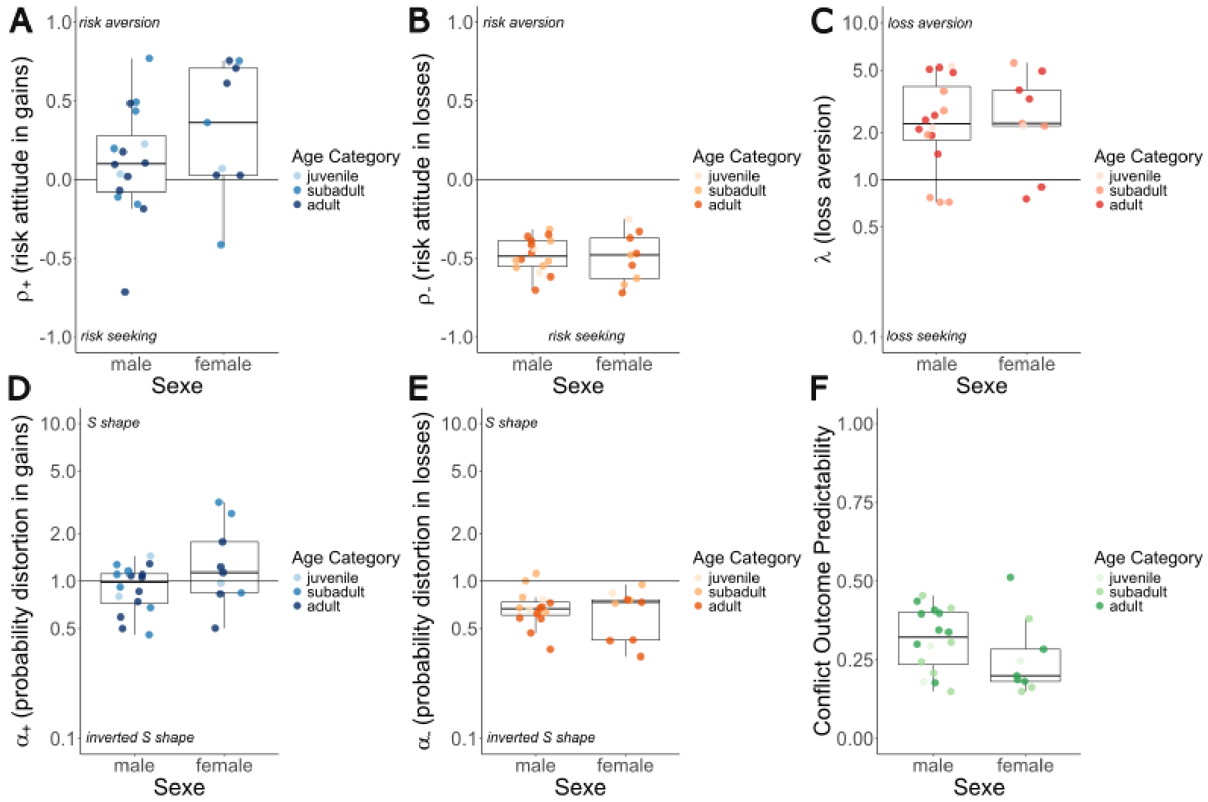

Supplementary Figure 8: Influence of sex on social and decision-making parameters. In all panels, each point represents the mean of all fitted social or decision-making parameters from 1,500 trial periods per individual. Darker points represent adults, while lighter points represent juveniles, with subadults shown in intermediate blue. Blue points correspond to PT parameters in the gain domain, whereas orange points correspond to parameters in the loss domain.

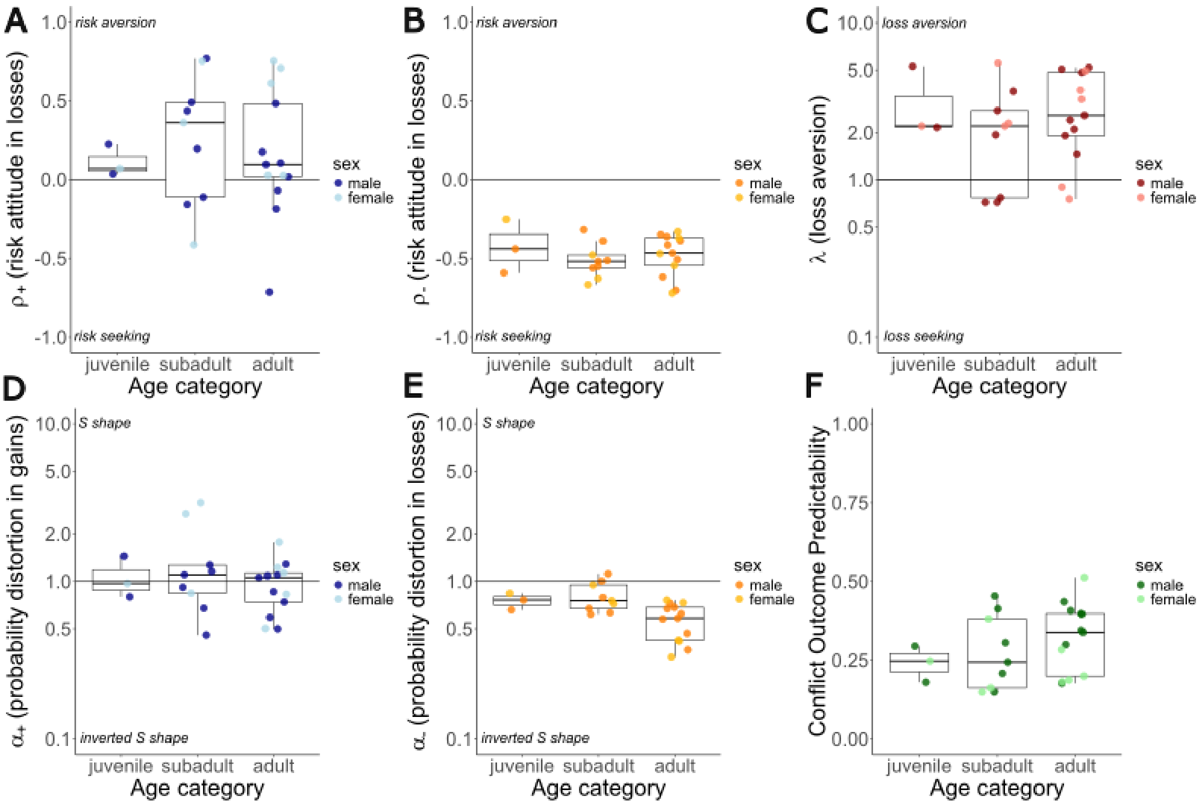

Supplementary Figure 9: Influence of age category on social and decision-making parameters. In all panels, each point corresponds to the mean of all fitted social or decision-making parameters from 1500 trial periods per individual. The darker points represent males, whereas the lighter points represent females. Blue points correspond to PT parameters in the gain domain, while orange points correspond to those in the loss domain.

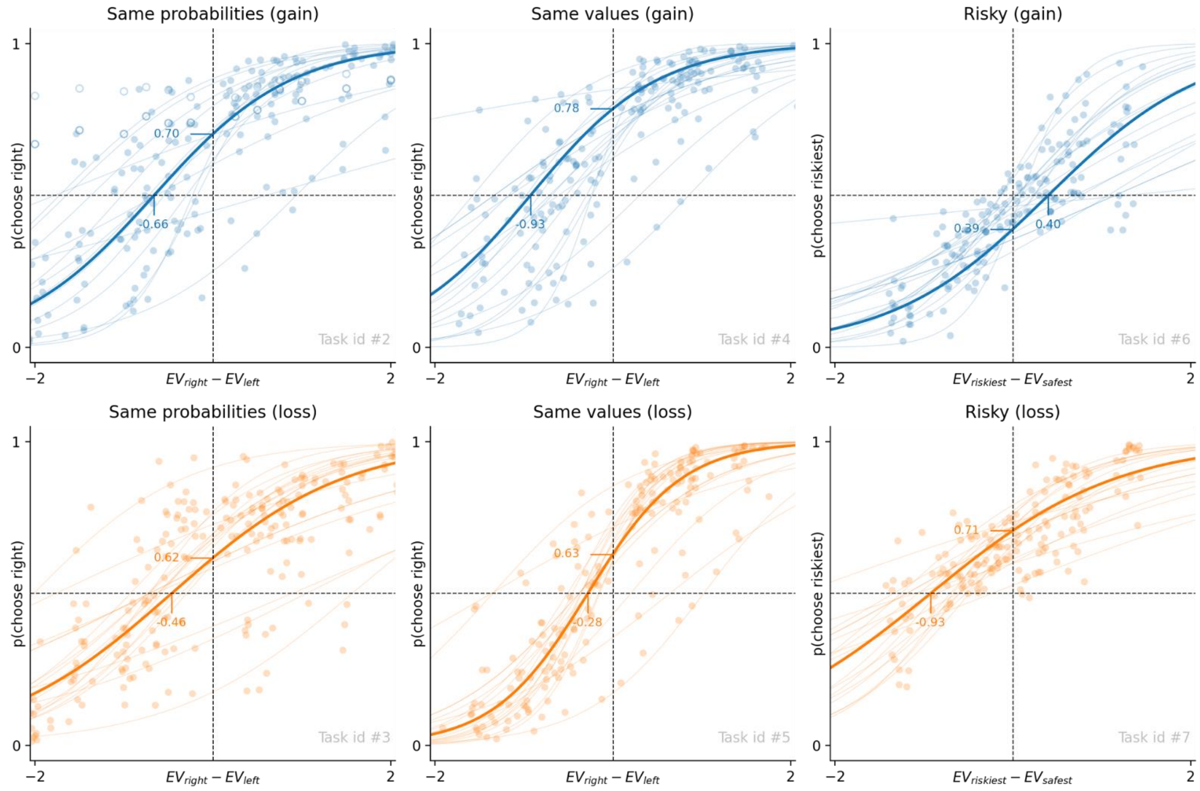

Supplementary Figure 10. Monkeys' decision-making across six lottery choice tasks. Each subplot examines consideration of expected value (EV) differences under different conditions: when probabilities are equal but amounts differ, when amounts are equal but probabilities differ and when there is a trade-off between quantity and probability. Blue lines (top row) represent choices involving gains, orange lines (bottom row) represent choices involving losses. Scattered points show individual monkey means binary choices (0/1) plotted against EV differences. Thin lines represent individual sigmoid fits (one line per monkey), while thick colored lines show the mean function across all monkeys. The panels in the first two columns show probability of choosing the option with higher expected value, while panels in the last column show probability of choosing the riskiest option.

Trials Summary by Sex and Age Category					
	**N**	**Total Trials**	**Trials per Individual**	**Trials per Day**	**Age**
**Females**					
Juvenile	1	9990	9990.0 ± NA	136.85 ± NA	3.6 ± NA
Subadult	3	122190	40730.0 ± 23866.3	151.59 ± 30.81	5.9 ± 1.3
Adult	5	277001	55400.2 ± 48784.3	146.10 ± 62.34	13.1 ± 6.1
**Males**					
Juvenile	2	39555	19777.5 ± 4635.1	185.91 ± 73.21	2.7 ± 0.1
Subadult	6	427862	71310.3 ± 68188.1	276.77 ± 89.87	6.3 ± 0.8
Adult	8	504230	63028.8 ± 51674.3	175.56 ± 76.53	8.3 ± 1.3

Supplementary Table 1. Summary of analyzed data by sex and age category of individuals. N represents the number of individuals per age and sex categories. A single individual may be represented in two distinct age categories if their age exceeds the limit of one category (see Materials and Methods).

	ρ^+			ρ^-			α^+			α^-			λ		
Predictors	*Estimates*	*CI*	*p*	*Estimates*	*CI*	*p*	*Estimates*	*CI*	*p*	*Estimates*	*CI*	*p*	*Estimates*	*CI*	*p*
(Intercept)	-0.04	-0.22 – 0.13	0.633	-0.53	-0.63 – -0.43	**<0.001**	0.15	-0.18 – 0.47	0.374	-0.46	-0.71 – -0.22	**<0.001**	1.32	0.92 – 1.73	**<0.001**
age category [juvenile]	-0.06	-0.26 – 0.14	0.575	0.16	0.05 – 0.27	**0.004**	-0.78	-1.12 – -0.44	**<0.001**	-0.09	-0.37 – 0.20	0.549	-0.58	-1.05 – -0.11	0.016
age category [subadult]	-0.06	-0.13 – 0.02	0.123	-0.01	-0.05 – 0.03	0.516	0.09	-0.04 – 0.21	0.165	0.04	-0.07 – 0.14	0.490	-0.18	-0.35 – -0.00	0.049
sex [female]	0.19	-0.07 – 0.44	0.149	0.01	-0.14 – 0.15	0.925	0.22	-0.25 – 0.70	0.359	-0.16	-0.51 – 0.19	0.379	-0.12	-0.71 – 0.46	0.677
mean rank	-0.01	-0.07 – 0.04	0.569	0.03	0.00 – 0.06	0.044	0.10	0.02 – 0.18	0.020	-0.01	-0.08 – 0.07	0.871	0.16	0.04 – 0.28	**0.009**
mean rank^2	0.09	0.06 – 0.11	**<0.001**	-0.00	-0.02 – 0.01	0.649	-0.13	-0.18 – -0.09	**<0.001**	-0.02	-0.06 – 0.02	0.397	-0.22	-0.28 – -0.15	**<0.001**
mean trial number	-0.20	-0.23 – -0.18	**<0.001**	0.01	-0.00 – 0.02	0.208	0.08	0.04 – 0.12	**<0.001**	-0.02	-0.06 – 0.01	0.164	0.30	0.25 – 0.35	**<0.001**
**Random Effects**															
σ^2	0.06			0.02			0.15			0.12			0.32		
τ_{00}	0.07 $_{monkey}$			0.02 $_{monkey}$			0.23 $_{monkey}$			0.12 $_{monkey}$			0.34 $_{monkey}$		
ICC	0.53			0.56			0.60			0.52			0.52		
N	17 $_{monkey}$			17 $_{monkey}$			17 $_{monkey}$			17 $_{monkey}$			17 $_{monkey}$		
Observations	873			873			873			873			873		
Marginal R^2 / Conditional R^2	0.358 / 0.699			0.045 / 0.578			0.141 / 0.654			0.026 / 0.530			0.190 / 0.612		

Supplementary Table 2: LMM models results for PT parameters for individuals with ordinal rank instead of Elo score

	ρ^+			ρ^-			α^+			α^-			λ		
Predictors	*Estimates*	*CI*	*p*	*Estimates*	*CI*	*p*	*Estimates*	*CI*	*p*	*Estimates*	*CI*	*p*	*Estimates*	*CI*	*p*
(Intercept)	0.05	-0.12 – 0.21	0.591	-0.54	-0.63 – -0.45	**<0.001**	-0.03	-0.34 – 0.28	0.840	-0.50	-0.71 – -0.29	**<0.001**	1.02	0.64 – 1.41	**<0.001**
age category [juvenile]	-0.02	-0.22 – 0.19	0.876	0.20	0.10 – 0.31	**<0.001**	-0.81	-1.14 – -0.47	**<0.001**	-0.07	-0.34 – 0.20	0.625	-0.64	-1.12 – -0.17	**0.008**
age category [subadult]	-0.09	-0.16 – -0.01	0.024	0.01	-0.03 – 0.05	0.522	0.14	0.02 – 0.26	0.027	0.06	-0.04 – 0.16	0.233	-0.13	-0.30 – 0.05	0.157
sex [female]	0.21	-0.05 – 0.47	0.112	0.02	-0.11 – 0.16	0.727	0.27	-0.21 – 0.75	0.276	-0.15	-0.47 – 0.17	0.357	-0.05	-0.64 – 0.55	0.878
COP	0.04	0.02 – 0.05	**<0.001**	0.02	0.01 – 0.03	**<0.001**	-0.04	-0.07 – -0.01	**0.007**	0.01	-0.02 – 0.03	0.593	-0.08	-0.13 – -0.04	**<0.001**
mean trial number	-0.21	-0.23 – -0.19	**<0.001**	0.01	0.00 – 0.02	0.029	0.07	0.04 – 0.10	**<0.001**	-0.01	-0.04 – 0.02	0.371	0.27	0.22 – 0.31	**<0.001**
**Random Effects**															
σ^2	0.06			0.02			0.15			0.11			0.33		
τ_{00}	0.07_{monkey}			0.02_{monkey}			0.25_{monkey}			0.11_{monkey}			0.37_{monkey}		
ICC	0.54			0.52			0.62			0.49			0.53		
N	18_{monkey}			18_{monkey}			18_{monkey}			18_{monkey}			18_{monkey}		
Observations	927			927			927			927			927		
Marginal R^2 / Conditional R^2	0.328 / 0.694			0.040 / 0.543			0.121 / 0.664			0.028 / 0.506			0.139 / 0.596		

Supplementary Table 3. LMM models results for PT parameters. All models were computed on all 1500 trials periods for all individuals after filtering data. The quadratic term of Elo-rating reported in TableS1 was replaced by our measure of COP. As suggested in Figure 3F, the quadratic term of Elo-rating is better explained when replaced by COP than dominance certainty. Values in bold represent significant effects ($p < 0.01$).
